# Comparative Results of Primary, Intermediate and Secondary Amputations

**Published:** 1864-05

**Authors:** H. L. Thomas


### CHRONICLE OF MEDICAL SCIENCE.

*Comparative Results of Primary, Intermediate and Secondary Amputations.* Translated from the French of LEGOUEST, by H. L. THOMAS, M. D.

The results of primary are more favorable than those of intermediary amputations, and the results of the latter less so than those of secondary. These approach pretty near to those which Malgaigne* has given the name of pathologic amputations, and which he considers far less dangerous than traumatic amputations; pathologic amputations being practised for chronic affections of the limb, and traumatic amputations for accidental lesions.

The observations of our predecessors upon the result of amputations practised at different stages have been, in part, confirmed by the experience of our late wars.

In the English army, the primary and intermediate operations practised in the Crimea, from the 1st of April, 1855, to the end of the war, were—

| | | Deaths. | Ratio per 100. |
|---|---|---|---|
| Primary operations | 600 | 175 | 25.3 |
| Intermediate operations | 89 | 38 | 42.7 |

In the hospitals of the Bosphorus, from the 26th of September to the 27th of November, 1854:

| | | Deaths. | Under treatment. | Cured. | Ratio per 100. |
|---|---|---|---|---|---|
| Primary operations | 154 | 18 | 40 | 96 | 11.6 |
| Intermediate operations | 65 | 42 | 7 | 16 | 64.6 |

The proportion of deaths following primary operations practised in the Crimea is greater than in the hospitals of the Bosphorus, where the operated upon were at once placed under the best conditions; it is not so great, on the contrary, as a consequence of intermediate amputations, because already, at the time these were done in the hospitals of the Bosphorus, these crowded establishments no longer presented conditions so favorable as the ambulances of the Crimea.

In the French army, all the amputations, not including those of the fingers and toes, amounted during the whole campaign to 4,467, divided thus:

| | | Deaths. | Pensions. | Ratio per 100. |
|---|---|---|---|---|
| Primary operations | 3,234 | 2,337 | 897 | 72.2 |
| Intermediate operations | 852 | 600 | 252 | 70.4 |
| Stage not determined | 381 | 194 | 187 | 51 |
| | 4,467 | 3,131 | 1,336 | |

Among these operations, the double operations numbered 120, with the following results:

| | | Deaths. | Pensions. | Ratio per 100 |
|---|---|---|---|---|
| Primary operations | 53 | 38 | 15 | 71.7 |
| Intermediate operations | 67 | 51 | 16 | 76,2 |
| | 120 | 89 | 31 | |

The primary operations practised in the English army give, on

* Archives Générales de Médecine, 3e série, t. xiii. et xiv.

the one hand, 25.3 deaths per 100; on the other, 11.6 deaths per 100; and the consecutive operations 42.7 and 64.6 deaths per 100. The advantage then is with the first over the second. On the contrary, in the French army, the primary operations give 72.2 deaths per 100, and the consecutive 70.4 deaths per 100. There is then a slight difference in favor of the latter. But it must be recollected that the operations in which the stage was not determined only give 51 deaths per 100; that it is very probable they were primary; and that, in adding them to the primary operations, they only present a mortality of 70 per 100. According to the figures, then, there would only be an insignificant difference between the primary and consecutive operations practised in the French army of the East. We will explain ourselves presently upon the large number of our losses among our operations, compared with those of the English army.

Lastly, the remarkable statistical facts of Malgaigne* upon the major operations practised in the hospitals of Paris, and the more recent researches of d'U. Trélat, appear to place beyond a doubt the advantage of pathologic over traumatic amputations.†

| | Amputations. | | Deaths. | Ratio per 100. |
|---|---|---|---|---|
| Malgaigne, | Pathologic | 343 | 176 | 51.3 |
| | Traumatic | 166 | 104 | 62.7 |
| U. Trélat, | Pathologic | 568 | 223 | 39.3 |
| | Traumatic | 476 | 261 | 55.6 |
| | Undetermined | 106 | 28 | 26.4 |

*General Results of Amputations.*—Some surgeons, and among those of highest authority, Malgaigne and Velpeau, while admitting amputations in indispensable cases, are disposed to practise primary amputations as seldom as possible, and are inclined to lay it down, as a general principle, that the efforts for preservation of the limb, in every case, does not expose the patient to a greater hazard of death than amputation.‡

This proposition cannot be accepted, in a general manner, in military surgery; the difference of result obtained from the field and from the civil hospitals is very great, and is due to the causes that we have enumerated above. We are forced to admit, that the general results of amputation do not afford a large amount of success; but it is but just to acknowledge that this partial comparative statistics of success after or without amputation, including those which we have given upon fractures of the thigh, treated conservatively or by amputation, are not yet sufficiently numerous to establish a law, and that so far they only present elements of solution to the question without settling it definitively.

The general mortality of amputations is very variable under different circumstances and according to the reports furnished by different surgeons; Boucher esimated that two-thirds of the amputations were fatal; Faure assures us that, after the battle of Fontenoy (1745), out of 300 amputations, only 30 or 40 were successful; Bilguer reduced to one or two the successful amputations practised during the seven years war (1756). Larrey,§ invoking all his souvenirs, after thirty years of war, thought he had saved the three-fourths of his amputations. A. Blandin, surgeon of the Republic, says that, with the nicest care, we may hope to save three-fifths of our operations.

These are merely expressions, without figures and beyond criticism. In the following table will be found details which, probably without being rigorously exact, are nevertheless more certain, because of the large numbers which go to make up their elements:

| | Operations. | Deaths. | Ratio per 100. |
|---|---|---|---|
| Naval battle before Brest, 1794—Fercoq, | 60 | 2 | 3.3 |
| Battle of Neubourg, 1794—Percey and Larrey, | 106 | 8 | 7.5 |
| Naval battle of Aboukir, 1798—*English army,* | 30 | – | – |
| Id.—French army, from Masselet, . . | 14 | 3 | 21.4 |
| Campaign of New Orleans, 1814—Guthrie, | 52 | 12 | 23.1 |
| Battle of Toulouse, 1814—Guthrie. . . | 99 | 32 | 32.3 |
| Battle of Waterloo, 1815—Guthrie, . . | 372 | 191 | 51,4 |
| Naval battle of Navarino, 1827—Del Signore, | 58 | 14 | 24.1 |
| Paris, Gros-Caillon, 1830—H. Larrey, . . | 17 | 9 | 53.0 |
| Paris, Hôtel-Dieu, 1837—Ménière, , . | 24 | 17 | 70,7 |
| Paris, Rouse, 1830, . . . . . . | 14 | 7 | 50.0 |
| Paris, Saint-Louis, 1832—Richerand, . . | 15 | 11 | 73.4 |
| Siege of Anvers, 1833—H. Larrey, . . | 64 | 14 | 21.9 |
| Spain, 1836-37—Alcocke, . . . . | 77 | 36 | 46.8 |
| Expedition of Constantine, 1837—Sédillot, | 23 | 17 | 73.9 |
| Paris, 1848—*Académie de Médicine*—(Divers), | 120 | 56 | 46.6 |
| Paris, 1848—Baudens, . . . . . . | 14 | 9 | 64.1 |
| Danish army, 1848-50—Djourp, . . . | 243 | 66 | 39.5 |
| Campaign of the East—*English army,* 1854-56, | 998 | 273 | 27.4 |
| Campaign of the East—*French army* (capital operations only), . . . . . . | 4,466 | 3,131 | 70.2 |
| Total, . . . . . | 6,797 | 3,916 | 57.63 |

The 6,797 operations collected in this table give the average of deaths as 57.63 per cent., a figure approaching very near the estimate of A. Blandin. An enormous difference exists between the ratios of mortality when taken separately. It was during the expediton of Constantine that the mortality was greatest, 73.9 per cent.; then comes, successively, that of Saint-Louis, in 1832, 73.4 per cent.; of the Hôtel-Dieu, in 1830, 70.7; of the French army during the Crimean campaign, 70.2 per cent.; of Val-de-Grace, in 1848, *Baudens*, 64.1; of Gros-Caillon, in 1830, *H. Larrey*, 53 per cent.; of the battle of Waterloo, *Guthrie*, 51.4; lastly, of Paris, 1830, *Rouse*, 50 per cent. The sad conditions to which the wounded were exposed during the disastrous expedition of Constantine, during the long war of the Crimea, after the fatigue of a battle of giants like that of Waterloo, in the crowded wards of Saint-Louis and the Hôtel-Dieu, the possible moral situation of wounded soldiers and citizens, victims of the street fights, appears to us sufficient to explain this great mortality.

The small mortality from operations, after certain engagements, Aboukir—English army 0 per cent;; naval battle before Brest, 3.3 per cent.; fight of Neubourg, 7.5 per cent.; Aboukir—French army, 21.4 per cent.; siege of Anvers, 21.9 per cent.; New Orleans, 23.1 per cent.; Navarino, 24.1 per cent.; appears to us more difficult to understand. It is a very remarkable fact that the operations practised after a naval battle give, generally, a smaller mortality than the others; perhaps, it may be attributed to portioning the wounded among a larger number of vessels; to the robust and hardened constitutions of the marines; to the absence of any disturbance of their habits—the vessel being for them a habitation and becoming the hospital after having been the field of battle. We will remark here, that the campaigns of Neubourg, New Orleans and seige of Anvers, which furnish the smallest mortality after operations, were short and light campaigns, during which the troops had not the time to become fatigued, and where they were surrounded with cares and resources without number. It is well to recollect that surgery is generally very successful at the beginning of a campaign; but practise it as the war is prolonged, upon men in less favorable conditions, and its success is more and more diminished.

This is the great cause of the afflicting mortality of our operations in the campaign in the East, 70.2 per cent., where inclemency of climate, epidemics and crowded hospitals were working their influence. The relatively small mortality of operations in the English army, our ally in this campaign, is surprising; the amount, 27.4 per cent., is nearly about the same as that estimated by Larrey after thirty years of war. The great success of English over French surgery manifests itself as well in civil as in military practice; it has so moved the surgeons on this side of the channel, that certain

* Archives Générales de Médecine, 3e serie, t. xiii. et xiv.

† Bulletin de l'Académie de Médecine, 1862.

English statistics, recently published in France, have been highly questioned, and considered as embodying manifest errors.

For our part, we accept the statistics given in the work entitled, "Medical and Surgical History of the British Army which served in Turkey and the Crimea, during the War with Russia, in the years 1854, '55, '56; London, 1858;" and presented in 1858 to the English Parliament. We will, nevertheless, remark: 1st. That the number of operations amounting to 998, and that of the deaths to 273, really reaches the number of 1,080 operations on the one hand, and of 310 deaths on the other, which is apparent on an analysis of the English statistical table; hence, it results that the mortality was 28.7 per cent. instead of 27.4. 2d. That 737 amputations under treatment, out of 1,080, were transferred from the Crimea and Scutasi to England, and that they are included among the number cured.

The difference of mortality, 1.3 per cent., between 27.4 per cent., figure of the original table, and 28.7 per cent. of the revised table, is not of enough importance to arrest our attention. But we cannot help noticing the uncertainty which hangs over the fate of the 737 amputations transferred to England and credited as cured. Did none of the patients succumb from the time of the transfer to 1856, the date at which the surgical history of the English campaign, as well as the war, ended? Did all the patients arriving at Chatham, numbering 667 out of the 737 transferred, survive the operation? We may legitimately doubt this, when it is remembered how many of the operated upon died during the transfer from the Crimea to Constantinople, and from Constantinople to France, and even afterwards, when it is known, moreover, how many fatal accidents supervene during the cicatrization and before the complete cure of an amputation. It is pretty certain that the authors of the English statistics have not included in their list of mortality, prematurely gotten up, the losses resulting from all the secondary and deferred accidents of amputations, and that they have thus given, without designing it, an average of mortality much lower than it really is; while, in the estimates relative to the French army, the result of the operations is not determined until the 31st of December, 1857; that is to say, eighteen months after the campaign; the long period embraced by these statistics comprehends the primary and consecutive results of the operations.

These considerations may modify to some extent the recorded success of English surgery in the East, but they do not explain the numerous reverses of our practice during the same campaign. Observations of a different order give us a more positive solution, and one of capital importance. The larger the army, the greater is its number of sick and wounded, and the greater also is its mortality and penury, in spite of the wisest precautions and the best directed foresight of administrators and surgeons. The effective force of the English army in the Crimea was never as great as ours. It did not reach beyond 97,864 men; ours was 309,000 men. Their effective force was almost wholly renewed, and was not completed until the spring of 1855; ours only received successive contingent additions. The number of English wounded by fire and sword by the enemy was only 12,164; ours amounted to 39,868.

In the discussion which took place in the Academy of Medicine in 1862*, to which the surgical statistics of the civil hospitals of London gave rise, a good deal was said about installation, hygienic arrangements, and the material and alimentary resources found in these institutions and represented as being much superior to those of our own. Personally, we are not in possession of any document upon this subject; but during the campaign of the East everybody had an opportunity of seeing the English ambulances and hospitals, poorly organized at first, rapidly ameliorated under the impulse of commissioners with full power sent from London to inspect them, and acquire, as well with regard to location, the bedding and clothing of the patients, as to the alimentary regime, a degree of comfort which our much more numerous establishments could not equal, despite the zeal, the devotion and the efforts of the medical and administrative staff. Let us add that the transfer of our patients from the Crimea periodically encumbered our hospitals at Constantinople, perpetuating purulent infection and hospital gangrene; while the English transfers, necessarily less numerous than our own, were almost all directed at once to the mother country; that the wounded and those operated upon of the English army, did not leave the Crimea until they were under way of cure, while those of the French wounded or operated upon were at once, or within a few days, sent away to make room for others succeeding them, without interruption. If transferring is an excellent measure, it is upon condition that it is only applied to men in conditions to be transferred: when the necessities of war oblige us to transfer indiscriminately, day or night, all the wounded and all operated upon, not only to avoid crowding, but also in order to be able to accommodate the fresh wounded, transfering must be attended with grievous results. It was impossible for us, in a military point of view, to retain upon the hostile soil of the Crimea a large number of wounded, who might have become a serious embarrassment to the command in case of retreat or of re-embarkment.

Every unprejudiced mind will find in these different conditions, imposed by events, the reasons for the difference in the results obtained. Perhaps it would be well to take into consideration, in order to embrace all the elements of this question, the influence exerted upon the success of operations by the race of men who bear them, their hygienic habits and their alimentation. It is a last resource for explaining, in a general manner, the results of English surgery, whose success is sometimes of akin to prodigy.

* Bulletin de l'Académie de Médecine, t. xxvii.

Comparative Results of Primary, Intermediate and Secondary Amputations, translated from the French of Legouest, by H. L. Thomas, M. D.

CONFEDERATE STATES

MEDICAL AND SURGICAL JOURNAL,

